# Enhancing 5G Small Cell Selection: A Neural Network and IoV-Based Approach

**DOI:** 10.3390/s21196361

**Published:** 2021-09-23

**Authors:** Ibtihal Ahmed Alablani, Mohammed Amer Arafah

**Affiliations:** 1 Department of Computer Engineering, College of Computer and Information Sciences, King Saud University, Riyadh 11543, Saudi Arabia; arafah@ksu.edu.sa; 2 Department of Computer Technology, Technical College, Technical and Vocational Training Corporation, Riyadh 11472, Saudi Arabia

**Keywords:** 5G, cell selection, IoV, machine learning, neural network, user association, small cell, Los Angeles, ITS

## Abstract

The ultra-dense network (UDN) is one of the key technologies in fifth generation (5G) networks. It is used to enhance the system capacity issue by deploying small cells at high density. In 5G UDNs, the cell selection process requires high computational complexity, so it is considered to be an open NP-hard problem. Internet of Vehicles (IoV) technology has become a new trend that aims to connect vehicles, people, infrastructure and networks to improve a transportation system. In this paper, we propose a machine-learning and IoV-based cell selection scheme called Artificial Neural Network Cell Selection (ANN-CS). It aims to select the small cell that has the longest dwell time. A feed-forward back-propagation ANN (FFBP-ANN) was trained to perform the selection task, based on moving vehicle information. Real datasets of vehicles and base stations (BSs), collected in Los Angeles, were used for training and evaluation purposes. Simulation results show that the trained ANN model has high accuracy, with a very low percentage of errors. In addition, the proposed ANN-CS decreases the handover rate by up to 33.33% and increases the dwell time by up to 15.47%, thereby minimizing the number of unsuccessful and unnecessary handovers (HOs). Furthermore, it led to an enhancement in terms of the downlink throughput achieved by vehicles.

## 1. Introduction

The 5G wireless network is a future technology which requires huge capacity, high reliability, massive connectivity, and ultra-low latency [1,2]. The Internet of Things (IoT), smart cities, intelligent transportation, and remote surgery are examples of emerging 5G applications [3,4,5,6]. Internet of Vehicles (IoV) is a special form of the IoT where vehicles are connected to the internet and they can transmit data [7,8]. The IoV communication has four different types, which are Vehicle-to-Vehicle (V2V) communication, Vehicle-to-Pedestrian (V2P) communication, Vehicle-to-Infrastructure (V2I) and Vehicle-to-Network (V2N) communication [9,10]. Intelligent transportation system (ITS) refers to a smart system that aims to enhance mobility and safety issues by integrating information and communication technologies into the transportation field [11,12]. The IoV will play a significant role in the future intelligent transportation system [13].

**Figure 1:**
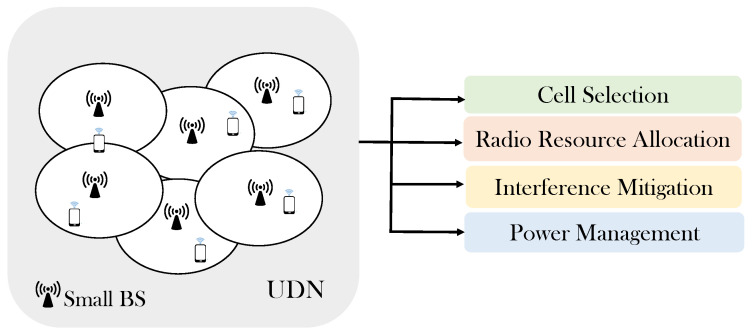
Main issues related to 5G ultra-dense networks.

An ultra-dense network is an enabling technology, which aims to meet the requirements of increased capacity and low latency [14]. It is a wireless network that has a high density of small cells that may exceed the number of active users [15]. However, UDNs have many challenges to be overcome, as illustrated in Figure 1. The main issues related to 5G UDNs are cell selection, radio resource allocation, interference mitigation, and power management [16,17,18]. Cell selection is the process of determining the small serving BS to which a mobile terminal will associate [19,20]. It is an NP-hard optimization problem and the computational complexity increases exponentially with increasing network size [14,21]. High data rates and the efficient use of a spectrum are crucial requirements for IoT-based 5G networks [22]. Maximizing the 5G data rate should be targeted so that the IoT transmission rate constraints and interference to IoT are considered. In addition, improving the energy efficiency by consuming less power is essential to meet communication requirements [23].

Nowadays, machine learning (ML) is becoming a promising method that can offer fast processing and real-time predictions for complex and large-scale applications by developing models and algorithms [24,25,26]. An artificial neural network (ANN) is a machine learning algorithm that is based on processing elements (called neurons) to simulate the concept of human neurons [27]. ANNs have proven their effectiveness in solving many problems in different fields [28]. Fifth generation (5G) networks require the application of machine learning techniques to operate effectively. Solving issues related to 5G wireless technology is an open direction for future research [29].

In this paper, we study the cell selection issue in 5G UDNs. A novel cell selection strategy is proposed that is based on ANN to perform the multi-classification task of small BSs, based on vehicle information. The main determinant in choosing a cell is the dwell time spent inside the cell. In the experiment, actual datasets are used for training and evaluation that were gathered in the city of Los Angeles.

The traditional scheme and most existing works give high priority to the small BSs that have the maximum received signal strength indicator (RSSI). However, relying on this principle is not effective in ultra-dense environments because it will lead to an increased handover rate [16,30,31]. In addition, machine learning techniques are needed to speed up processing time and to reduce computational complexity.

The main contributions of this work are:

1. proposing an intelligent ANN-based cell selection strategy for 5G UDNs, called ANN-CS. It aims to select a small BS that has the longest dwell time in the range, using a ML technique. A feed-forward back-propagation ANN (FFBP-ANN) was trained based on real BS and vehicle datasets that were collected in the city of Los Angeles;
2. evaluating the performance of the trained FFBP-ANN in terms of accuracy, sensitivity, specificity, precision, F-score, and geometric mean (G-mean). In addition, errors are checked based on the root mean square error (RMSE) and the mean absolute error (MAE);
3. evaluating the performance of the proposed ANN-CS scheme based on the following performance metrics: the average (i) dwell time; (ii) number of handovers; (iii) number of unsuccessful and unnecessary handovers; and (iv) achievable downlink throughput. Then, the performance of the proposed ANN-CS approach is compared with the traditional cell selection method and a recent related approach called Handover based on Residence Time Prediction (HO RTP).

The rest of this paper is structured as follows. Section 2 presents related ML-based cell selection works. The proposed machine-learning-based approach is explained in Section 3. The simulation results are discussed in Section 4. The conclusion of the whole paper and suggestions for future work are given in Section 5. Appendix A gives lists of all abbreviations and symbols that are mentioned in this paper.

## 2. Related Work

In this section, recent related user association methods are discussed. Some of these works use machine learning (ML) techniques to solve the cell selection issue, while others do not.

### 2.1. Non ML-Based Cell Selection Strategies

A cell selection approach was proposed by Kiishida et al. in [32] for 5G multi-layered Radio Access Networks (RANs). It considers the direction and velocity of UE movement to reduce the number of frequent handovers. The final decision is based on the value of SINR, whereby the BS that has the maximum SINR value will be selected. Simulation results proved that the proposed approach achieved an approximate 30% improvement in the number of handovers while maintaining the average flow time.

In [33], Elkourdi et al. proposed a cell selection algorithm for 5G heterogeneous networks that based on Bayesian game. There are two players, that is, user equipment (UEs) and access nodes (AN). There are different types of UEs based on the traffic. Simulation results showed that the proposed scheme outperformed the traditional and cell-range-expansion (CRE) methods in terms of the probability of proper connection and end-to-end delay.

Waheidi et al. developed an approach called Cell Association, based on a Multi-Armed Bandit game (CA-MAB) in [30]. There are two classes of devices, that is, UE and IoT, and the proposed CA-MAB scheme was evaluated in static and mobile environments. The evaluation results showed that the CA-MAB approach enhance the energy efficiency and the throughput and the existence of mobility affected the energy savings, throughput, and equilibrium.

Arshad et al. proposed topology-aware skipping approaches in [34], where various skipping techniques are considered. The handover decision is taken based on the position of a user and/or cell size. Simulation results showed that the proposed schemes outperformed the conventional RSSI-based method by up to 47% in terms of the average user throughput.

Two cell selection strategies for HUDNs were proposed by Sun et al. in [35] that depend on the coordinated multipoint (CoMP) technology. The first scheme is called movement-aware CoMP handover (MACH), which select the cooperation BSs set that has the strongest received signal with a dwell time greater than a specific threshold. The second scheme is known as improved MACH (iMACH), which adds the nearest BS to the MACH’s cooperation BSs set, instead of the BS that has the lowest RSSI value in the set. The handover is triggered based on MACH, when the farthest BS in the set becomes the nearest one. Conversely, in iMACH, the HO is initiated when the nearest BS becomes the farthest one. Simulation results demonstrated that MACH and iMACH strategies enhanced the average achievable throughput. In addition, they improve the coverage probability and handover rate.

Qin et al. introduced a cell selection strategy for 5G ultra-dense networks in [36]. It is called Handover based on Resident Time Prediction (HO RTP) and it aims to estimate the residence time inside a cell and select the base station that has the strongest RSSI value with a residence period longer than a predefined threshold. Simulation results demonstrated that the HO RTP approach was superior to the traditional method in terms of achievable mean user throughput.

In [16], Alablani and Arafah introduced an adaptive cell selection approach for 5G Heterogeneous UDNs (HUDNs), called ADA-CS. It aims to select the best BS based on the different features of HUDNs and vehicle movements. It passes through six phases to achieve its goals; namely, configuration, decision-making, filtering, narrowing, selecting, and HO triggering. Simulation results demonstrated that the ADA-CS strategy was superior to the conventional and recent related approaches in terms of the average number of handovers, average achievable downlink data rates and spectral efficiency.

### 2.2. ML-Based Cell Selection Strategies

In [37], Dilranjan et al. proposed a BS prediction strategy for 5G wireless networks that uses a Recurrent Neural Network (RNN) classifier. Received Signal Strength (RSS) values are used to train the RNN model. Simulation results showed that the proposed scheme achieved 98% accuracy in predicting the optimal base station to be associated with.

Zhang et al. introduced a machine-learning-based cell selection scheme for drones in wireless networks in [38]. A conditional random field (CRF) model is used to predict the best serving cells depending on signal-to- interference-plus-noise ratio (SINR) values. Simulation results demonstrated that the proposed CRF-based method yielded 90% accuracy in predicting the best cells and it outperformed two simple heuristic methods.

In [39], Perez et al. proposed a machine-learning-based framework to solve the user association problem in 5G heterogeneous networks. The Q-learning algorithm was used to achieve the model goal. 3-dimensional feature vectors were used that included the BS identification (BSID) index, downlink (DL) SINR, and the DL cell load. Simulation results showed the superiority of the proposed framework over alternative decision methods.

Zappone et al. introduced a user association method in [40] that was based on machine learning. A feed-forward artificial neural network (ANN) was trained to perform the optimal user association where the input was the geographical positions of users. The use of the ANN reduced the computational complexity of the assignment procedure compared to conventional methods.

In [41], a cell selection issue was solved by introducing two hidden Markov-model- (HMM) based ML strategies that were proposed by Balapuwaduge et al. The reliability and availability of network resources were the main targets of the proposed HMM-based ML schemes. Simulation results showed the superiority of the proposed strategies compared with a random cell selection method in terms of channel availability and reliability.

An intelligent machine-learning-based user association for 5G heterogeneous networks was developed in [14] by Zhang et al. The problem was treated as a supervised learning task and a cross-entropy algorithm was used for labeling the best base station to be associated with. A U-Net convolutional neural network (CNN) was trained to solve the user association problem under the cell load constraint. Channel gain matrices were mapped onto images to be the inputs of CNN, while the user association matrices were the outputs of the ML model. Simulation results demonstrated that the proposed schemes enhanced computation time and network robustness.

Table 1 represents a comparison among recent ML-based cell selection schemes in terms of the ML model used and its inputs. Based on the cell selection works that are represented in this section, we found the following limitations:

- The number of cell selection schemes that rely on applying machine learning technologies in predicting the serving BS is small compared with the number of non ML-based works. However, using ML techniques seems to be essential in an environment that has vehicle movement and ultra-high density BSs to decrease the computational complexity of estimating the best BSs;
- Few works consider the estimation of the dwell time, which is, in fact, the main determinant in selecting BSs. Moreover, these works did not give the dwelling period a high priority compared to the value of the received signal strength. In addition, the equations used to estimate the dwell time are inaccurate and assume that the user is located at the edge of the cell, which is contrary to reality;
- The ML-based works did not give the model enough types of inputs to be able to predict the best BS efficiently.

## 3. Proposed ML-Based Cell Selection Strategy

### 3.1. Problem Formulation

The proposed ML-Based cell selection, the ANN-CS scheme, aims to reduce the handover rate in 5G UDNs by prolonging the dwell time of vehicles within small cells. Millimeter-Wave (mmWave) communication in UDNs has been considered, which operates in a high-frequency band. The association in downlink with single connectivity between small BSs and vehicles is considered. The small BSs located in the Central cluster of Los Angeles are denoted by $B_{\mathrm{small}}=\left\{B_{1},B_{2},\ldots ,B_{K}\right\}$. The vehicles, which move with different movement-related information, are represented by $V=\{V_{1},V_{2},\ldots ,V_{J}\}$. The BS association vector is expressed as $A=\{a_{11},a_{12},\ldots ,a_{\mathrm{KJ}}\}$, where the BS association variable that indicates the connection between small BS k and vehicle j is defined as shown in Equation (1).

(1) $$\begin{array}{c} a_{\mathrm{kj}}=\left\{\begin{array}{cc} 1 & \mathrm{If}\;\mathrm{there}\;\mathrm{is}\;\mathrm{association}\;\mathrm{between}B_{k}\;\mathrm{and}\;V_{j}\\ 0 & \mathrm{Otherwise} \end{array}\right. \end{array}$$

### 3.2. Proposed Framework

**Figure 2:**
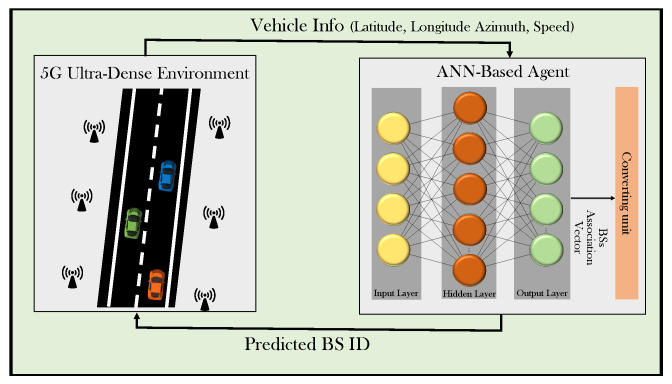
The framework of the proposed ANN-CS scheme.

The framework of the proposed ANN-based small cell selection is represented in Figure 2. The framework is composed of two main components: a 5G ultra-dense environment and an ANN-based agent. In training and testing processes, there is an interaction between the two components. The vehicle-related information, which includes geographical locations, azimuths, and speeds, is entered in the ANN-based agent. The ANN is used to predict the best small BS to be associated with, based on the longest dwell time, by generating BS-association vectors. A converting unit is used to convert the predicted BS association vector to the corresponding BS’s ID.

The pseudocode for the proposed ANN-CS scheme is shown in Algorithm 1.
**Algorithm 1:** Pseudocode for the proposed ANN-CS approach
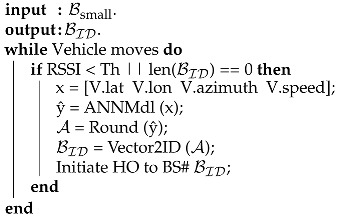


### 3.3. 5G Network Model

A 5G ultra-dense network has been modeled in this paper based on real datasets. In the city of Los Angeles, the distribution of small BSs is in three clusters: (a) Burbank, (b) Central, and (c) Long Beach [42]. The Central cluster is considered due to the high density of the small BSs.

**Figure 3:**
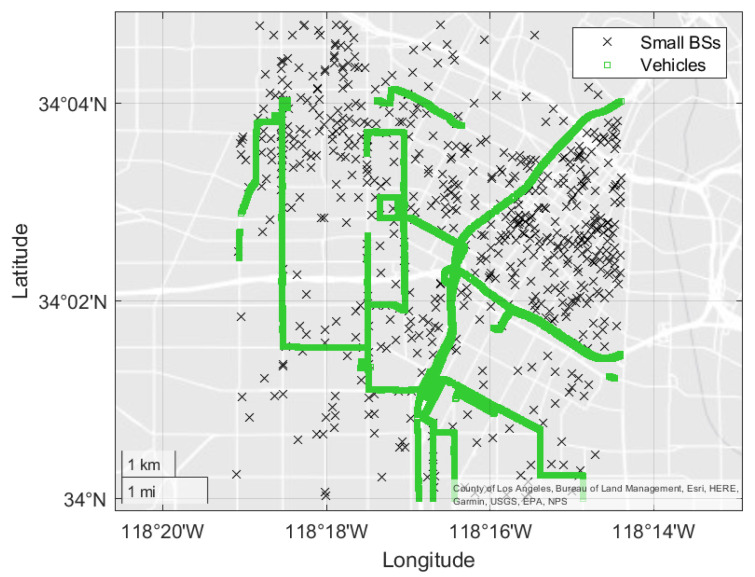
The system model in the city of Los Angeles.

The system model is shown in Figure 3, where the black crosses represent the distribution of small BSs and the series of green squares shows the locations of vehicles in LA City. There are 621 small base stations and 48,864 vehicles.

### 3.4. Machine Learning Model

**Figure 4:**
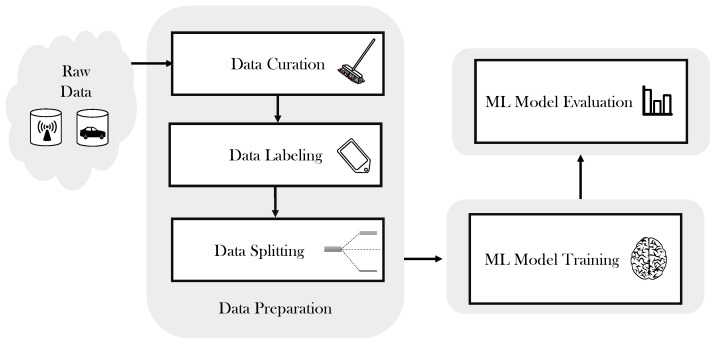
Building phases of the proposed ANN-based model.

This study is based on using a machine learning technique to solve the 5G small cell selection issue. There are three main phases involved in building the proposed ML model, as represented in Figure 4. These phases are (1) data preparation, (2) ML model training, and (3) ML model evaluation. The raw data consist of two databases; a dataset of small base stations located in the city of Los Angeles [42], and a dataset of vehicle information, which was collected in LA City [43].

#### 3.4.1. Data Preparation

**Figure 5:**
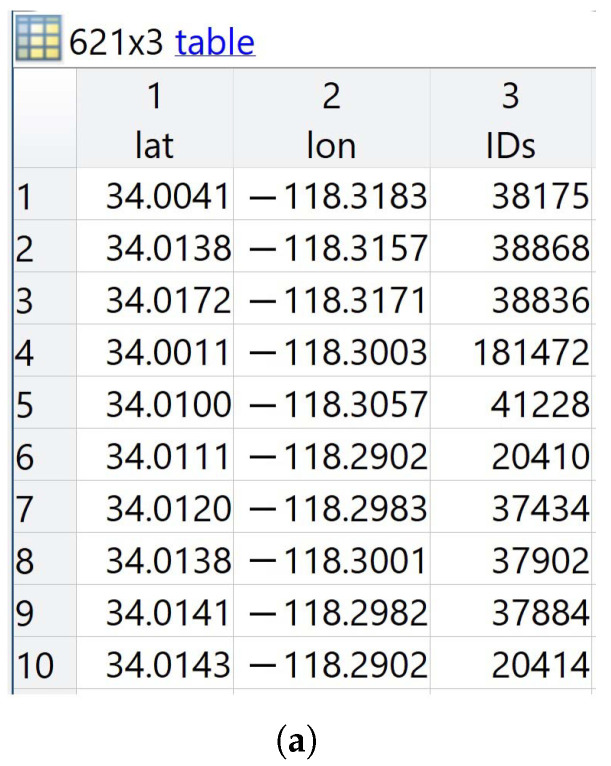

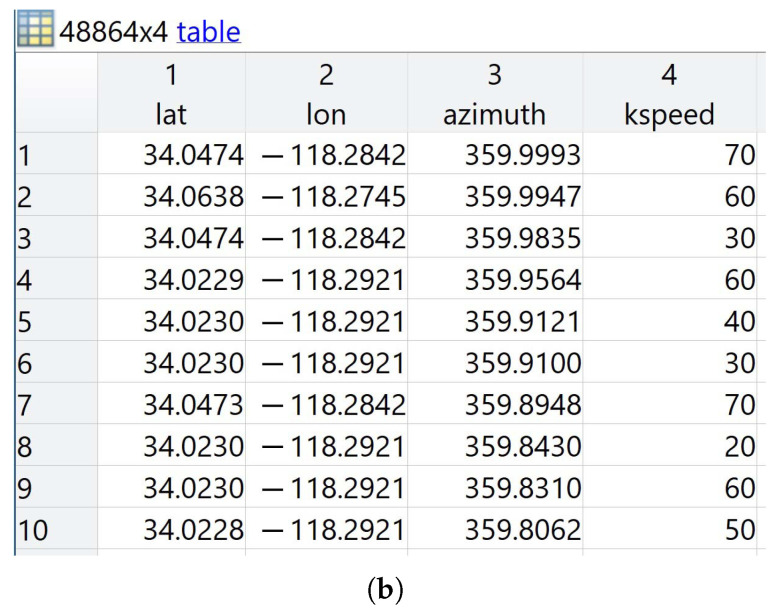
Snapshots of LA small BSs and vehicles tables after data curation step. (**a**) Snapshot of LA small BSs table. (**b**) Snapshot of LA vehicles table.

The data preparation phase is composed of three steps:

- **Data curation step:** the collected data are organized and information that does not serve the proposed ML model is cleared up in this step. From the LA small BS and vehicle datasets, the samples corresponding to the Central cluster area of LA were taken, due to the high density of small cells. In addition, the columns that are used in calculating the longest dwell time within each small cell are kept. Figure 5 shows snapshots of LA small BS and vehicle tables after the data curation step, where the number of small base stations and vehicles are 621 and 48,864, respectively. The LA small BS table has three columns; latitude (lat), longitude (lon), and the identification numbers of the small BS (IDs). The LA vehicles table has four columns; lat, lon, azimuth, and kspeed, where azimuth is the angle between the vehicle direction and the north in degrees. The kspeed is the speeds of the vehicles, which are randomly assigned in the range from 10 to 80 km/h.
- **Data labeling step:** This is a process of tagging LA vehicles data samples to solve a multi-classification problem via supervised learning. It is performed by generating a BS association vector for each vehicle, where 1 is assigned to the small BS that has the longest dwell time and 0 to the other BSs. The dwell time of a vehicle within small cells, $T_{\mathrm{dwell}}$, is estimated as represented in Equation (2).
  (2) $$\begin{array}{cc} T_{\mathrm{dwell}} & =\frac{C}{s}\\ \; & =\frac{d\;\cos\left(\theta \right)+\sqrt{r^{2}-d^{2}\;\mathrm{si}n^{2}\left(\theta \right)}}{s}, \end{array}$$
  where C is the chord of a small cell, which indicates the length of the dwelling distance within the small cell. The vehicle speed and the distance between the vehicle and small BS are identified by s and d, respectively. The angle between the small BS and the direction of the vehicle is represented by θ and r is the radius of the small cell.
- **Data splitting step:** The labelled data were split into training and testing sets with percentages of 80% and 20%, respectively. Table 2 shows the number of training and testing samples that are used to train and evaluate the proposed ANN-based model.

#### 3.4.2. ML Model Training

**Figure 6:**
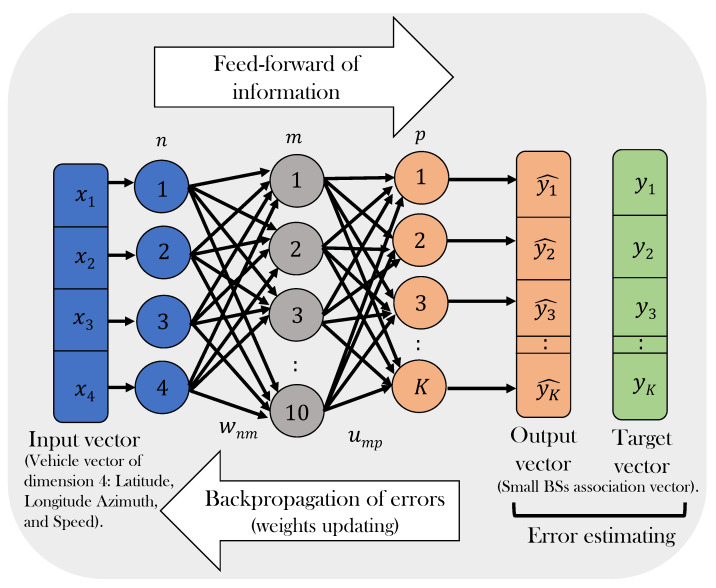
Illustration of the proposed neural network architecture.

A feed-forward back-propagation ANN (FFBP-ANN) is used to achieve the multi-classification task, as shown in Figure 6. In the proposed FFBP-ANN structure, there are three layers; input, hidden, and output. The input vector has four values related to vehicles; latitude, longitude, azimuth, and speed. The training data set contains 39,091 feature vectors with different vehicle information. The hidden layer is composed of ten neurons, while the output layer includes K neurons to generate the small BSs association vector. Based on the target vector, the errors are estimated to update the weights of the proposed neural network. Table 3 shows the training parameters that were used for training the proposed FFBP-ANN model.

#### 3.4.3. ML Model Evaluation

The root mean square error (RMSE) is a common measure, which calculates the error distance between the predicted values. The mean absolute error (MAE) is a measure used to compute the average of the absolute difference between the predicted and the target values. RMSE and MAE are defined as shown in Equations (3) and (4), respectively [44].

(3) $$\begin{array}{c} \mathrm{RMSE}=\sqrt{\frac{1}{N}\;\Sigma_{i=1}^{N}{\left({\hat{y}}_{i}-y_{i}\right)}^{2}} \end{array}$$

(4) $$\begin{array}{c} \mathrm{MAE}=\frac{1}{N}\;\Sigma_{i=1}^{N}\left|y_{i}-{\hat{y}}_{i}\right|, \end{array}$$

where the number of testing samples is denoted by N and the predicted and the target small BSs are represented by $\hat{y}$ and y, respectively.

To evaluate the performance of the proposed ANN-based model, a confusion matrix is constructed, which is sometimes called a contingency table [45]. The confusion matrix is an effective tool that reports the numbers of true positive (TP), true negative (TN), false positive (FP), and false negative (FN) [46]. Based on the constructed confusion matrix, accuracy, sensitivity, specificity, precision, F-score, and geometric mean (G-mean) are calculated as defined in Equations (5)–(10).

(5) $$\begin{array}{c} \mathrm{Accuracy}=\frac{\mathrm{TP}+\mathrm{TN}}{\mathrm{TP}+\mathrm{TN}+\mathrm{FP}+\mathrm{FN}} \end{array}$$

(6) $$\begin{array}{c} \mathrm{Sensitivity}=\frac{\mathrm{TP}}{\mathrm{TP}+\mathrm{FN}} \end{array}$$

(7) $$\begin{array}{c} \mathrm{Specificity}=\frac{\mathrm{TN}}{\mathrm{FP}+\mathrm{TN}} \end{array}$$

(8) $$\begin{array}{c} \mathrm{Precision}=\frac{\mathrm{TP}}{\mathrm{TP}+\mathrm{FP}} \end{array}$$

(9) $$\begin{array}{c} \mathrm{F-score}=\frac{2\mathrm{TP}}{2\mathrm{TP}+\mathrm{FP}+\mathrm{FN}} \end{array}$$

(10) $$\begin{array}{c} \mathrm{G-mean}=\sqrt{\left(\mathrm{Sensitivity}\times \mathrm{Specificity}\right)}. \end{array}$$

### 3.5. Propagation Channel Model

The parameters used in the propagation channel model, that is, path loss (PL), fading and shadowing, are shown in Table 4. The PL model used is the 3rd Generation Partnership Project (3GPP) model, which is given by the 3GPP technical report (specification #38.901, version 16.1.0) [47]. Urban microcell-line-of-sight (UMi-LOS)/street canyon model is considered in this study. In the Central cluster of LA city, streets are flanked by buildings on both sides, resulting in canyon-like environments, and the small BSs are shorter than the buildings. The path loss function, γ(d), is associated with the distance between a small base station and a vehicle, where the distance (d) is measured in meters and the carrier frequency ($f_{c}$) is expressed in GHz. The breakpoint distance is represented by $d_{BP}$ and the height and the effective height of the small BS are denoted by $h_{B}$ and $h_{B}^{{}^{\prime }}$, respectively. The height and the effective height of the vehicle are expressed as $h_{V}$ and $h_{V}^{{}^{\prime }}$. The velocity of light in free space is represented by c. The Rayleigh fading model is a common model that can represent multipath fading in real-world environments [48,49]. In this work, multipath fading is modeled as Rayleigh fading to represent the LA city environment, which follows the exponential distribution with unit mean. In this paper, frequency-selective fading is not considered because measurements made in [50] demonstrate that the delay spread is generally small. Moreover, using techniques like orthogonal frequency-division multiplexing (OFDM) or frequency domain equalization limits the effect of the frequency-selectivity in fading [51]. In addition, small-scale fading at mmWave cellular systems is less severe than that in Long-Term Evolution (LTE) systems when using base station antennas with narrow beams, as the measurement results show [52]. The log-normal shadowing is included in the propagation model, where $\sigma_{SF}$ is the shadow-fading standard deviation in decibels (dB).

## 4. Simulation Results and Discussion

In this work, the MATLAB simulator 2021a was the simulation tool used to implement and analyze the performance of the proposed ANN-CS algorithm. A high-performance gaming computer was used to perform the data processing and to evaluate the performance. The specifications of the computer are given in Table 5.

### 4.1. Evaluation of the Trained ANN Model

**Figure 7:**
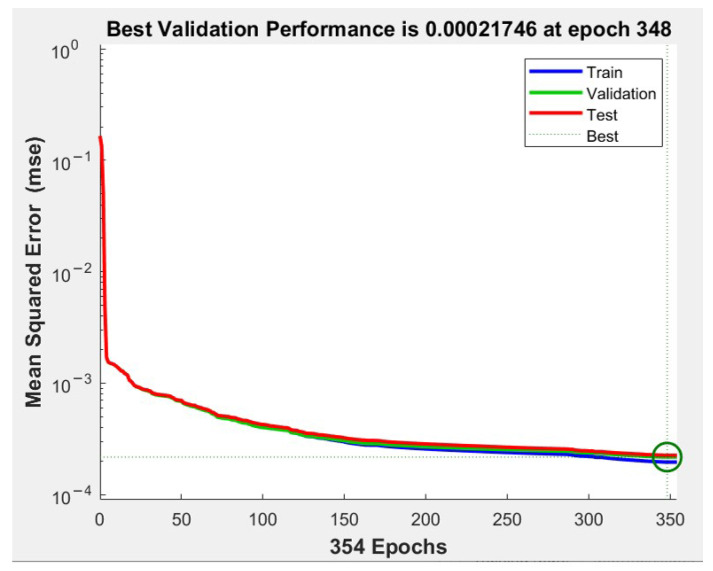
The relation between number of epochs and training MSE.

Figure 7 illustrates the validation performance chart that shows the relations between the number of epochs and the mean squared error (MSE). In the chart, there is a green circle indicating the training stopping time, which occurs when the validation error reaches its minimum and then increases at epoch 354. The test set and the validation set are represented by green and red lines. For all training, validation and test data, as the number of epochs increases, the MSE value decreases. The best training MSE of the trained network equals 0.00021746, which was obtained at epoch 348, and is very small.

**Figure 8:**
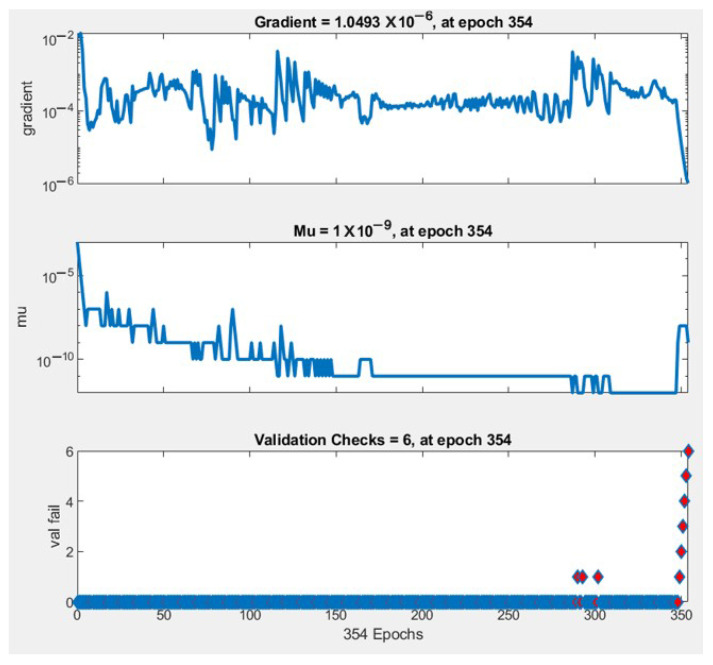
The relations between the number of epochs and the performance of the training state.

Figure 8 illustrates the relations between the number of epochs and the performance of the training state in terms of gradient, Mu factor, and validation fail. The values of the gradient, Mu factor, and validation check at epoch 354 are 1.0493 $\times \;10^{-6}$, 1$\;\times \;10^{-9}$ and 6, respectively.

The trained ANN-based model was evaluated based on RMSE, MAE, accuracy, sensitivity, specificity, precision, F-score, and G-mean, as shown in Table 6.

### 4.2. Evaluation of the Proposed ANN-CS Scheme

#### 4.2.1. Performance Metrics

The performance of the proposed ANN-CS strategy is evaluated in terms of:

- **Average dwell time:** The average dwell time of a vehicle in a small cell is estimated according to Equation (11), where the number of moving vehicles in an ultra-dense network is expressed as J.
  (11) $$E\left(T_{\mathrm{dwell}}\right)=\frac{\sum_{J}\left(\sum \left(T_{\mathrm{dwell}}\right)/N_{HO}\right)}{J}.$$
- **Average number of handovers:** The average number of HOs that occurs as vehicles move in the UDN is computed according to Equation (12).
  (12) $$E\left(N_{\mathrm{HO}}\right)=\frac{\sum_{J}N_{\mathrm{HO}}}{J}.$$
- **Average number of unsuccessful HO:** An unsuccessful HO occurs when the handover latency is longer than the dwell time within a small cell ($l_{i}$) [53]. The probability of an unsuccessful HO (Prα) can be calculated in terms of vehicle speed (s), small cell radius (r), handover latency (l), and the time threshold of an unsuccessful HO ($Th_{\alpha }$), as shown in Equation (13). Equation (15) shows the formula to estimate the average number of unsuccessful HOs ($E(N_{\alpha })$).
  (13) $$Pr\left(\alpha \right)=\left\{\begin{array}{cc} \frac{2}{\pi }\left[\arcsin\left(\frac{sl_{i}}{2r}\right)-\arcsin\left(\frac{sTh_{\alpha }}{2r}\right)\right] & 0⩽Th_{\alpha }⩽l_{i}\\ 0 & l_{i}<Th_{\alpha } \end{array}\right.$$
  (14) $$Th_{\alpha }=\frac{2r}{s}\sin\left(\arcsin\left(\frac{sl_{i}}{2r}\right)-\frac{2}{\pi }Pr\left(\alpha \right)\right)\;\;\;;0⩽Pr\left(\alpha \right)⩽1$$
  (15) $$E\left(N_{\alpha }\right)=Pr\left(\alpha \right)\times E\left(N_{HO}\right).$$
- **Average number of unnecessary HOs:** An unnecessary HO means a false handover is performed, where the dwell time in a small cell is shorter than the summation of HO latencies to move into ($l_{i}$) and out ($l_{o}$) of the small cell [54]. The probability of an unnecessary HO (Pr(β)) can be calculated as expressed in Equation (16). The time threshold of the unnecessary handover is denoted by $Th_{\beta }$. Equation (18) illustrates the method of computing the average number of unnecessary HOs ($E(N_{\beta })$).
  (16) $$\begin{array}{c} Pr\left(\beta \right)=\left\{\begin{array}{cc} \frac{2}{\pi }\left[\arcsin\left(\frac{s(l_{i}\;+\;l_{o})}{2r}\right)-\arcsin\left(\frac{sTh_{\beta }}{2r}\right)\right] & 0⩽Th_{\beta }⩽\left(l_{i}\;+\;l_{o}\right)\\ 0 & \left(l_{i}+l_{o}\right)<Th_{\beta } \end{array}\right. \end{array}$$
  (17) $$Th_{\beta }=\frac{2r}{s}\sin\left(\arcsin\left(\frac{s(l_{i}\;+\;l_{o})}{2r}\right)-\frac{2}{\pi }Pr\left(\beta \right)\right)\;\;\;;0⩽Pr\left(\beta \right)⩽1$$
  (18) $$E\left(N_{\beta }\right)=Pr\left(\beta \right)\times E\left(N_{HO}\right).$$
- **Average achievable DL throughput:** The purpose of deploying a high density of 5G small cells is to provide a high data capacity with a cost-effective method [55]. The achievable DL data rate of vehicles during movement in UDNs is calculated according to Shannon’s equation, as expressed in Equation (19). The signal-to-interference-plus-noise ratio (SINR), which is denoted by $\zeta_{kj}$, is the ratio of the received signal to the interference from other wireless BSs plus noise [56].
  (19) $$R_{kj}=W\;\;log_{2}\left(1+\zeta_{kj}\right)$$
  (20) $$\begin{array}{c} \zeta_{kj}=\frac{p_{tx_{k}}\gamma_{kj}\left(d\right)H_{kj}}{\sum_{i\neq k}\left(p_{tx_{k}}\gamma_{ji}\left(d\right)H_{ji}\right)+\sigma^{2}},\\ \;\;\forall \;V_{j}\in V\;\mathrm{and}\;\forall \;B_{k}\in B_{\mathrm{small}}. \end{array}$$
  The maximum transmission power of small BSs is denoted as $p_{tx}$ and the path loss function is represented by γ(d), which is defined in Section 3.5. The channel gain is expressed as H, which includes the effects of Rayleigh fading and log-normal shadowing. The thermal noise ($\sigma^{2}$) is modeled as an additive white Gaussian noise (AWGN), as shown in Equation (21). It can be computed in terms of noise power spectral density ($N_{0}$), and sub-channel bandwidth (W).
  (21) $$\sigma^{2}=N_{0}W.$$
  A throughput is the sum of effective achievable data rate over the network during movement [35]. The throughput of a vehicle can be calculated based on Equation (22).
  (22) $$\mathrm{Throughpu}t_{j}=\sum_{k}R_{kj}\;\;\forall \;B_{k}\in B_{\mathrm{small}}.$$

#### 4.2.2. Performance Results

In this section, we compare the performance of our proposed ANN-CS approach with the traditional and HO RTP cell selection schemes. The simulation parameters that are used in this work are listed in Table 7.

**Figure 9:**
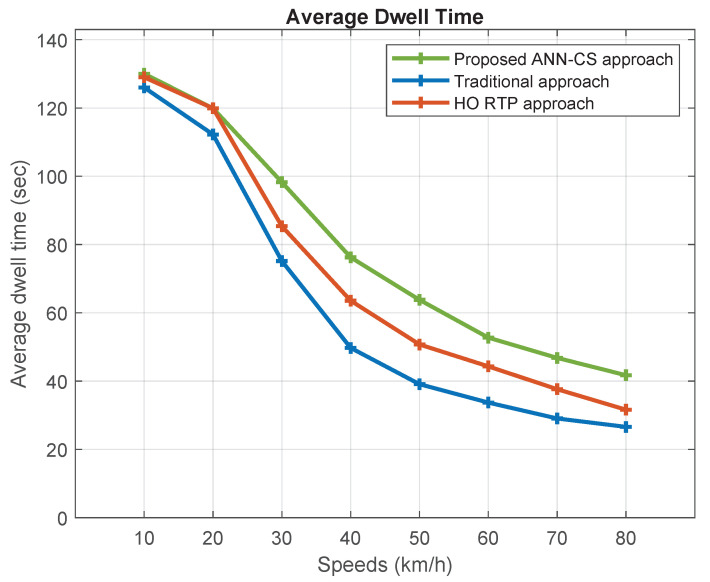
Average dwell time.

**Figure 10:**
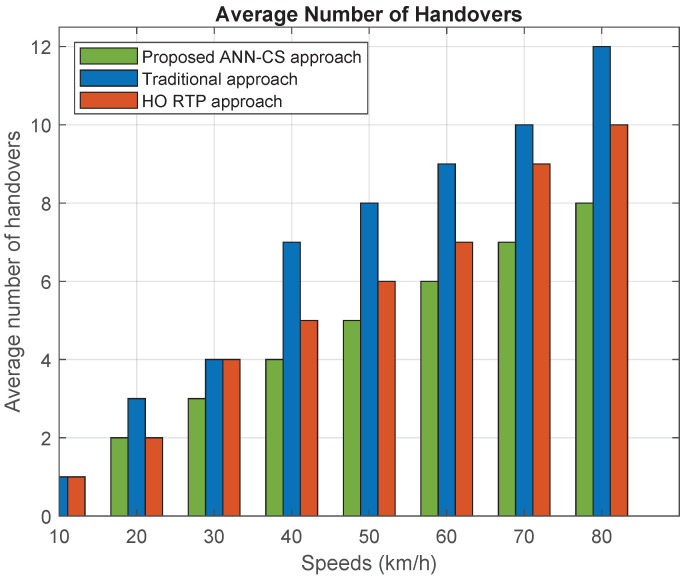
Average number of HOs.

Figure 9 and Figure 10 represent the average dwell time and average number of handovers under different moving speeds. Increasing the speed will reduce the average dwell time of vehicles inside small cells and, therefore, increase the average number of handovers. The proposed ANN-CS approach prolongs the dwell time by estimating it based on the direction and speed of vehicles in addition to small cell specifications. As the chart indicates, the ANN-CS approach has the longest average dwell time and it is superior to the traditional and HO RTP approaches by 15.47% and 7.56%, respectively. The reason is that the traditional cell selection method chooses the small BS that has the largest RSSI value, even if it does not lie on a vehicle’s trajectory. The ANN-CS strategy outperforms the HO RTP approach because HO RTP estimates the time resident inside the cell but it selects the small BS that has the highest signal strength value with residence time greater than a predefined dwell time threshold. Therefore, the primary criterion for selection is the strength of the received signal. In addition, the ANN-CS approach outperforms the traditional and RTP HO schemes by 33.33% and 18.18% in terms of the average number of handovers.

**Figure 11:**
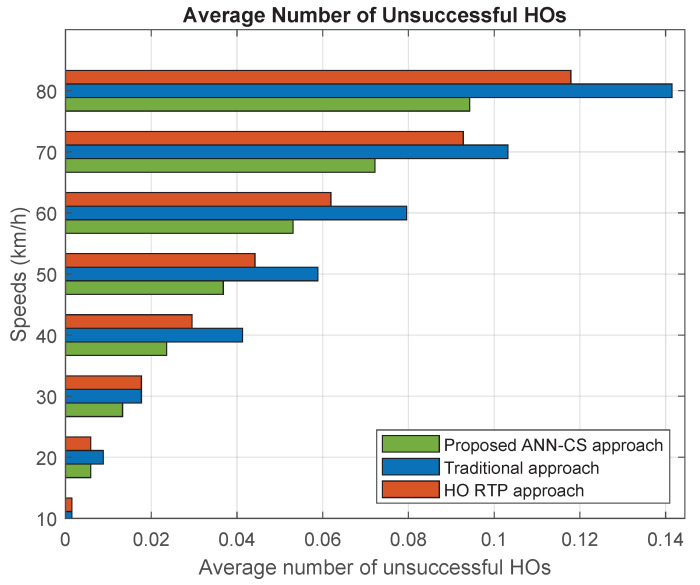
Average number of unsuccessful HOs.

**Figure 12:**
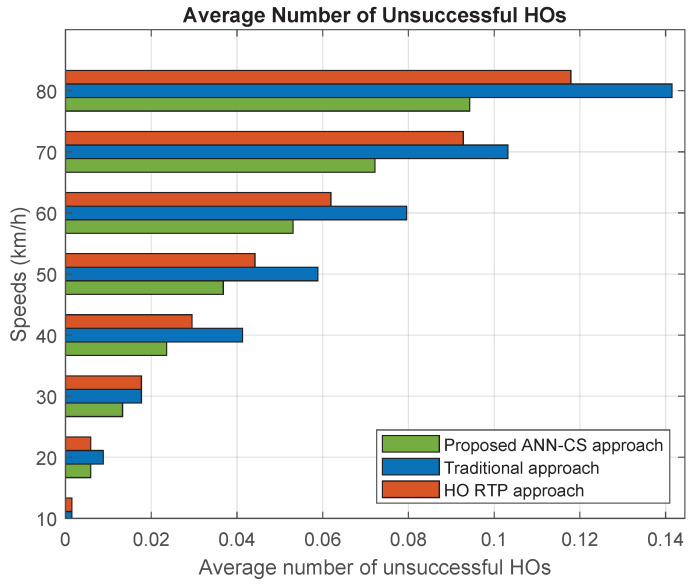
Average number of unnecessary HOs.

Figure 11 and Figure 12 represent the average number of unsuccessful and unnecessary handovers against different vehicle speeds. Increasing the speed of vehicles leads to an increase in the probabilities of unsuccessful and unnecessary HOs due to the decrease in the length of the dwelling period inside the small cell. In terms of the average number of unsuccessful and unnecessary handovers, our proposed ANN-CS approach outperforms the traditional and HO RTP selection schemes by 33.55% and 19.04%, respectively.

**Figure 13:**
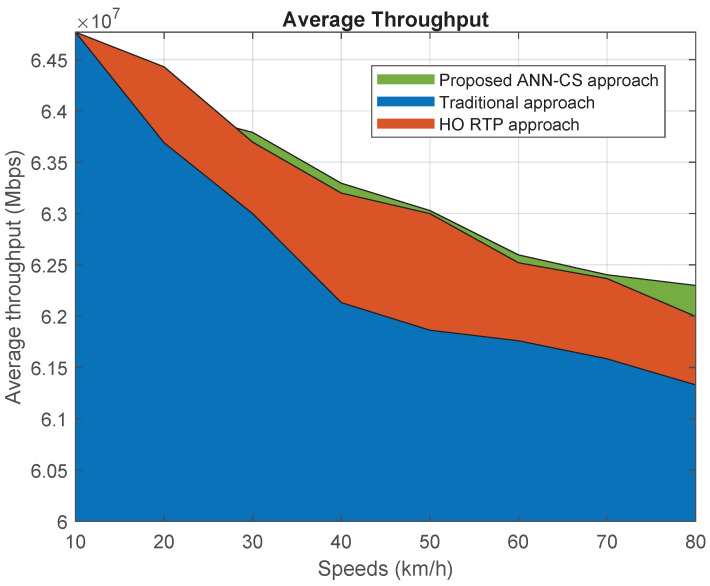
Average achievable downlink throughput.

Figure 13 displays the relationship between the average achievable downlink throughput and vehicle speed. We found that the proposed ANN-CS made improvements over the traditional and HO RTP approaches by 1.2% and 0.1%, respectively. Although the ANN-CS method does not choose the closest small cell, it can achieve enhancements over the methods that give high priority to the received signal strength criteria. This is because the achievable DL throughput is negatively affected by an increase in the number of HOs due to the latency caused by moving from one small cell to another. In addition, the peak data rate is usually reached by our ANN-CS scheme when the vehicle is at the middle of the small cell, while the peak data rates may not be achieved by RSSI-based methods.

## 5. Conclusions and Future Work

The IoV is a fundamental technology that will improve the transportation system. In ultra-dense networks, cell selection is considered an NP-hard problem. In this paper, we solve the cell selection issue for 5G UDNs by applying a machine learning technique. A neural network and IoV-based algorithm called the ANN-CS scheme is proposed that uses a trained feed-forward back-propagation ANN model to perform the multi-classification task of selecting small base stations. It aims to prolong the dwell time within small cells and thereby decrease the number of handovers. Real datasets are used for training and evaluation purposes, which were collected in the city of Los Angeles. The trained ANN-FFBP model is able to predict the best small BS with high accuracy and a very low error percentage. Simulation results show that our proposed ANN-CS scheme can achieve its goals by decreasing the HOs rate and prolonging the dwell time of vehicles within small cells, and thus the numbers of unsuccessful and unnecessary HOs are minimized. Moreover, the achievable DL throughput is enhanced when using our approach compared with other existing methods. In addition, the computational complexity is reduced by using the ANN, compared with non-ML-based methods. For future work, other machine learning techniques can be applied to solve the cell selection issue in 5G UDNs. A machine learning model can be trained based on different types of input features to make the model applicable to different environments.

## Figures and Tables

**Table 1:** A comparison among recent ML-based cell selection schemes.

Ref	Year	Authors	ML Model	ML Inputs	Model Performance
[37]	2017	Dilranjan et al.	RNN	RSS values	Achieves high accuracy of 98%
[38]	2017	Zhang et al.	CRF	SINR values	Achieves high accuracy of 90%
[39]	2017	Juan et al.	Q-Learning	3D feature vectors formed by: (1) BSID index. (2) DL SINR. (3) DL cell load	Enhances load balancing
[40]	2018	Zappone et al.	ANN	Geographical positions of users	Reduced computational complexity
[41]	2019	Balapuwaduge et al.	HMM	Initial HMM and observation sequence	Improves channel availability and reliability
[14]	2020	Zhang et al.	U-Net CNN	Channel gain matrices	Enhances computation time and network robustness

**Table 2:** Number of training and testing samples.

	Training Set	Testing Set
**Number of samples**	39,091	9773

**Table 3:** The training parameters.

Training Features	Training Parameters
Neural network type	Feed-forward backprop
Number of layers	3 (Input, Hidden, and Output)
Number of hidden layer neurons	10
Activation functions	tansig
Initial weights	[0–1]
Number of iterations	1000
Number of epochs	1
Learning rate	0.01
Training time (days)	14

**Table 4:** The parameters used in the propagation channel model.

Parameter	Model	Formula
Path loss	3GPP UMi-LOS (street canyon)	$\gamma (d)=\{\begin{array}{cc} 32.4+21\;log_{10}\left(d\right)+20\;log_{10}\left(f_{c}\right) & 10\;m⩽d⩽d_{BP}\\ 32.4+40\;log_{10}\left(d\right)+20\;log_{10}\left(f_{c}\right)-9.5\;log_{10}\left({\left(d_{BP}\right)}^{2}+{\left(h_{B}-h_{V}\right)}^{2}\right) & d_{BP}⩽d⩽5\;km \end{array}$
		where
		$d_{BP}=4\;h_{B}^{{}^{\prime }}\;h_{V}^{{}^{\prime }}\;f_{c}/c$
		$h_{B}^{{}^{\prime }}=h_{B}-h^{{}^{\prime }}$
		$h_{V}^{{}^{\prime }}=h_{V}-h^{{}^{\prime }}$
		$h^{{}^{\prime }}=1$ m
		$c=3\times 10^{8}$ m/s
Fading	Rayleigh fading (unit mean)	$H_{\mathrm{Rayeigh}}∽\exp\left(1\right)$
Shadowing	Log-normal	$H_{\mathrm{Normal}}∽N\left(\sigma_{SF}\right)$

**Table 5:** Gaming computer specifications.

Component	Feature
Processor	AMD Ryzan 7 3800X 8-Core Processor @3.89 GHz
Memory	64 GB DDR RAM
GPU	NVIDIA EVGA GeForce RTX 2070 Super
Motherboard	ASRock B450M Pro4
Power Supply	Gamemax 800 W
Hard Disk	SSD 3 TB
Cooling System	Corsair H100i v2
Operating System	Windows 10 64-bit

**Table 6:** The trained model evaluation values.

Performance Metrics	Value
RMSE	0.0157
MAE	0.00024683
Accuracy (%)	99.9039
Sensitivity (%)	88.8571
Specificity (%)	99.9518
Precision (%)	88.8571
F-score (%)	88.8571
G-mean (%)	94.2413

**Table 7:** Simulation parameters.

Simulation Parameter	Value
Number of 5G small BSs	621
Vehicle height (m)	1.8
Small BS height (m)	10
RSSI threshold (dBm)	−90
Carrier frequency (GHz)	28
System bandwidth (MHz)	500
Transmission power (Watt)	1
Shadowing standard deviation (dB)	4
Thermal noise density (dBm/Hz)	−174
Handover latency (s)	1
Target value of Prα	0.02
Target value of Prβ	0.04
Simulation time (s)	350

## Appendix A. Lists of Abbreviations and Symbols

Lists of the abbreviations and symbols that are used in this paper are given in Table A1 and Table A2, respectively.

**Table A1:** List of abbreviations.

Abbreviations	Full Term
3GPP	Third Generation Partnership Project
5G	Fifth-Generation
AN	Access Node
ANN	Artificial Neural Network
ANN-CS	Artificial Neural Network Cell Selection
AWGN	Additive White Gaussian Noise
BS	Base Station
BSID	Base Station Identification
CA-MAB	Cell Association based on Multi Armed Bandit game
CNN	Convolutional Neural network
CoMP	Coordinated Multipoint
CRE	Cell Range Expansion
CRF	Conditional Random Field
DL	Downlink
FFBP-ANN	Feed-Forward Back-Propagation ANN
G-mean	Geometric mean
HMM	Hidden Markov Model
HO	Handover
HO RTP	Handover based on Resident Time Prediction
iMACH	improved MACH
IoT	Internet of Things
IoV	Internet of Vehicles
LA	Los Angeles
MAE	Mean Absolute Error
MACH	Movement-Aware CoMP Handover
ML	Machine Learning
PL	Path Loss
RMSE	Root Mean Square Error
RAN	Radio Access Network
RNN	Recurrent Neural Network
RSSI	Received Signal Strength Indicator
SINR	Signal-to-Interference-plus-Noise Ratio
UDN	Ultra-Dense Network
UE	User Equipment
UMi-LOS	Urban Microcell-Line-Of-Sight
V2I	Vehicle-to-Infrastructure
V2N	Vehicle-to-Network
V2P	Vehicle-to-Pedestrian
V2V	Vehicle-to-Vehicle

**Table A2:** List of main symbols.

Symbol	Description
J	Number of vehicles
K	Number of small BSs
j	Index of vehicles
k	Index of small BSs
B	All small BSs
V	All moving vehicles
A	Small BSs association vector
a	Association variable between a vehicle and a small BS
$T_{\mathrm{dwell}}$	Dwell time of a vehicle inside a small cell
$N_{HO}$	Number of handovers
C	Length of small cell’s chord
s	Speed of a vehicle
d	Distance between a small BS and a vehicle
θ	Angle between small BS and the direction of a vehicle
r	Radius of a small cell
R	Achievable downlink data rate
x	Input data
y	Target data
$y^{\prime }$	Predicted data
γ(d)	Path loss associated with distance d
ζ	SINR of a vehicle received from small BSs
$d_{BP}$	Breakpoint distance
$h_{B}$	Height of small BS
$h_{B}^{{}^{\prime }}$	Effective height of small BS
$f_{c}$	Carrier frequency
$h_{V}$	Height of a vehicle
$h_{V}^{{}^{\prime }}$	Effective height of vehicle
$\sigma_{SF}$	Shadow fading standard deviation
H	Channel gain
$l_{i}$	HO latency to move into small cell
$l_{o}$	HO latency to move out of small cell
Pr(α)	Probability of unsuccessful HOs
Pr(β)	Probability of unnecessary HOs
$Th_{\alpha }$	Time threshold of unsuccessful HOs
$Th_{\beta }$	Time threshold of unnecessary HOs
$p_{tx}$	Maximum transmission power of small BS
$\sigma^{2}$	The thermal noise
$N_{0}$	Noise power spectral density
W	Sub-channel bandwidth
